# The Impact of Particle Size and Surface Treatment of Zirconia Suspension for Photocuring Additive Manufacturing

**DOI:** 10.3390/ma16041670

**Published:** 2023-02-16

**Authors:** Mee-Jin Jun, Jin-Ho Kang, Kumaresan Sakthiabirami, Seyed Aliakbar Hosseini Toopghara, Ye-Seul Kim, Kwi-Dug Yun, Sang-Won Park

**Affiliations:** 1 Department of Dental Hygiene, Gwangju Health University, Gwangju 62287, Republic of Korea; 2 Department of Prosthodontics, School of Dentistry, Chonnam National University, Gwangju 61186, Republic of Korea; 3 Biomedical Evaluation and Research Centre, School of Dentistry, Chonnam National University, Gwangju 61186, Republic of Korea; 4 Department of Medical Engineering Joint Research, Chonnam National University, Gwangju 61186, Republic of Korea

**Keywords:** additive manufacture, dispersion stability, silane coupling agent, viscosity, zirconia suspension

## Abstract

To prepare a photocurable ceramic suspension for use in commercialized additive manufacturing equipment, the effects of the rheological properties of zirconia particles added to a binder, and the presence or absence of a silane coupling agent on the particles was evaluated. To this end, three experimental groups (ZSs, ZMs, ZLs) and three control groups (ZS, ZM, ZL) were designed depending on the size of the underlying zirconia particles. The test-group zirconia suspensions were prepared through silanization, which was not applied to the control-group suspensions. Depending on the particle size, viscosity differences between the test and control groups were 16,842, 18,623, and 12,303 mPa·s, respectively. Compared to the other groups, the viscosity of the ZLs group suspension decreased by 70.98–88.04%. This confirmed that the viscosity of the suspensions was affected by the particle size and the presence of silane coating. The dispersion stability of the zirconia suspensions was evaluated over 20 days. A sedimentation test confirmed that the sedimentation rate of the ZLs group was slower than those of the other groups. This study aimed to optimize the suspension manufacturing method to effectively be utilized in further commercializing zirconia three-dimensional (3D) printing and could also help to develop various medical applications.

## 1. Introduction

Zirconia is an oxide ceramic with excellent mechanical properties, and it can overcome the problems of low strength and low hardness of the existing ceramic prosthesis materials. For this reason, zirconia has attracted considerable interest in the field of dentistry because of its esthetic appearance with a similar color to that of human teeth [1,2,3].

Over the years, zirconia prostheses have been manufactured using several methods, including the cutting of pre-sintered and fully sintered zirconia by using CAD/CAM (computer-aided design/computer-aided manufacturing) or CM (copy-milling) systems [4,5]. Zirconia prostheses processed using CAD/CAM systems may have rough scratches or defects on their surfaces, which eventually develop into cracks after sintering and may reduce their strength [1,6,7]. For this reason, in the dental field, additive manufacturing-type three-dimensional (3D) printing technology has recently been introduced as a manufacturing process that provides high precision. Photopolymerization-based technology (photocuring additive manufacturing) employs a liquid solution that is solidified owing to the photocuring reaction of a photosensitive resin under ultraviolet (UV) irradiation. Successive layers are stacked on top of the last layer [8,9,10]. In addition, with photocuring additive manufacturing, it is possible to achieve high precision, excellent surface quality, and high printing speeds [11,12,13,14,15,16].

**Figure 1:**
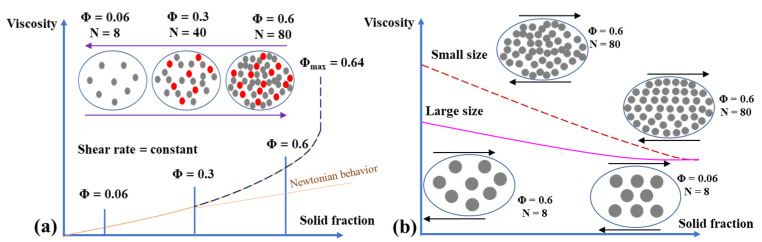
(**a**) The viscosity of sphere-bearing suspensions versus solid fraction under a constant shear rate and constant sphere size. (**b**) Shear-rate dependence of small and large spheres for a continuous solid fraction (Grey spheres indicate the particle, whereas red spheres denote the particle collision which occurs due to increased solid fraction and arrows indicate the shear stress).

To ensure that the prostheses manufactured using the zirconia additive manufacturing method exhibit clinically applicable physical performance, a high content of zirconia particles (40–60 vol.%) must be included in the binder [15]. Here is where the problem arises: as the zirconia particle content increases, the viscosity, light scattering, and refractive index of the suspension increase; and the dispersion, stability, and fluidity of the particles in the suspension decrease, which adversely affect the 3D printing process and act as an obstacle for obtaining better mechanical properties. [17,18,19,20,21,22,23,24,25]. As the fraction of solid particles added to the binder increases, the possibility of collisions between the particles increases, and these interparticle collisions hinder particle movement in the suspension. During particle collision, friction adds additional shear force, which leads to an increase in viscosity (Figure 1a). When the solid fraction reaches its maximum value (φmax), the force required to shear the sample increases significantly owing to interparticle interactions and exceeds the required shear stress [26]. At certain solid fraction values, when the interparticle interactions intensify, shear thinning is observed. The viscosity difference between small and large particles decreases at higher shear rates because the particles are rearranged favorably in the flow direction (Figure 1b) [26]. Suspensions containing spheres of both sizes exhibit shear thinning; however, the phenomenon is more pronounced in the suspension prepared using the smaller spheres.

To improve the behavior of and solve the problems associated with zirconia suspensions that contain a high fraction of solid particles, studies are being conducted to achieve low viscosity while maintaining a high particle content by subjecting the particles to surface treatment using a silane coupling agent [27,28,29,30,31,32,33]. In general, a large number of hydroxyl groups are present on the surface of ceramic particles; and, as a result, these particles have hydrophilic properties, and they tend to aggregate [34]. The silane coupling agent transforms the hydrophilic surface of ceramic particles into a hydrophobic surface. The hydrophobic anchoring head group and hydrophilic end chain create a steric barrier that ensures uniform dispersion of particles in suspensions [35]. For this reason, silane coupling agents modify the surface of ceramic particles and increase their dispersion stability [20], improve their interfacial adhesion, and facilitate the preparation of high-volume-fraction suspensions. The aim of the present study is to optimize the suspension’s poor rheological properties through identifying the factors that affect zirconia suspensions with a silane coupling agent in various particle sizes for possible utilization in further photocurable additive manufacturing or other techniques. In addition, the poor rheological properties of the high-content zirconia suspensions were enhanced, which would positively affect the 3D printing process.

## 2. Experimental Details

### 2.1. Experimental Material

In this experiment, three types of zirconia powders (TZ-3Y, TZ-3YS-E, Tosho, Anjo Shi, Japan; TZY-3, Qingdao Terio Corporation, Qingdao, China) were used to prepare a zirconia suspension (Table 1). Methyltrimethoxysilane (MTMES, SAMCHUN Chemical, Seoul, Korea) was used as a silane coupling agent to modify the surfaces of the zirconia powders. The suspension was prepared using a UV-curable resin as the base, which was a mixture of 1,6-Hexanediol diacrylate (HDDA, Sigma Aldrich Inc., St. Louis, MO, USA), photocurable resin (XYZPRINTING rigid, New Taipei City, Taiwan), photoinitiator (Irgacure 819, Ciba Specialty Chemicals, Basel, Switzerland), and a dispersant called Disperbyk (BYK-180, BYK, Chester, NY, USA).

### 2.2. BET Analysis

In BET analysis, the surface of the samples meets a gas at a series of pressures that will absorb the surface and the walls of the pores in the samples. Various gases could be used; however, nitrogen is the most common absorbate. The reason is that N_2_ is a diatomic non-spherical, and it has a quadrupole moment. This will lead it to preferentially absorb to the surface and remain for longer. So, the surface area and pore volume of the powders were analyzed by conducting N_2_ adsorption/desorption measurement at 77.3 K by using a surface area and pore-size analyzer (BELSORP-mini II, Microtracbel, Osaka, Japan).

### 2.3. Particle Size Analysis and Zeta Potential Measurement 

We used DLS )Zetasizer NanoZS90, Malvern Instrument Ltd., Worcestershire, UK) to measure and compare the particle size and surface charge of the zirconia particles. Dynamic Light Scattering (DLS), is a precise, non-invasive, fast, reliable, technology that is well established for size measurement and study of the size distribution of particles in submicron sizes. Samples were dispersed in ethanol and 1 mL of each sample was transferred to a quartz glass cuvette. The measurements were performed thrice, and the averages of the three values were used in this study.

### 2.4. Silane-Coated Zirconia Powder Manufacturing

**Figure 2:**
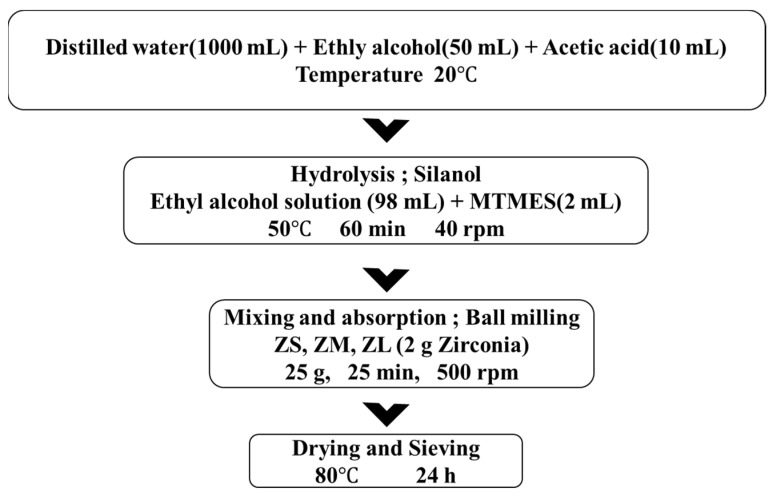
Flowchart of powder modification and suspension.

To apply silane coating on the selected zirconia particles (ZS, ZM, ZL), distilled water and ethyl alcohol were mixed in a ratio of 20:1. Acetic acid (64-19-7, Daejung Chemicals Co., Ltd., Busan, Republic of Korea) was added and stirred into this solution until the solution pH was 4, as measured using a pH meter (OROION STAR A21, ThermoFisher, Waltham, MA, USA). MTMES (2 mL) was added to the prepared pH 4 solution and stirred at 40 rpm for 60 min at 50 °C to form silanol. Approximately 25 g of each of the three zirconia powders of various particle sizes was added to 50 mL of the silanol solution and stirred at 500 rpm for 25 min to achieve homogeneous mixing. Then, the solutions were dried for 24 h at 80 °C in a drying oven (p-6 Planetary Mill, Fritsch, Co., Ltd., Idar-Oberstein, Germany). Thereafter, the dried zirconia powders were classified using a 100 µm sieve, and the final silane-coated zirconia powders (ZSs, ZMs, ZLs) were obtained. This experiment was subdivided into six groups: three control groups (ZS, ZM, ZL) and three experimental groups (ZSs, ZMs, ZLs). A flowchart of powder modification with silane is presented in Figure 2.

### 2.5. Fourier-Transform Infrared Spectroscopy (FT-IR)

This technique uses IR radiation to determine the chemical structures of the samples. When the radiation passes through the sample, some radiation will absorb and some of them will be transmitted. This spectrum acts like a fingerprint to determine the chemical structures. The chemical structures of the zirconia particles included in the control and experimental groups and the presence of silane coating on them were determined in the range of 450–5000 cm^−1^ using Fourier-transform infrared spectroscopy (FTIR, Spectrum 400, Perkin Elmer, Waltham, MA, USA).

### 2.6. Observation of Surface-Treated Zirconia

We used a transmission electron microscope (field-emission transmission electron microscope, JEM-2100F, JEOL Ltd., Akishima, Japan) to observe and compare the coating morphologies of MTMES on the surfaces of the nanoparticles in the experimental and control groups.

### 2.7. Slurry Preparation

Zirconia photocuring suspensions were prepared with a volume fraction of 40 vol.% by using zirconia powders from the three experimental and three control groups. MTMES was added to suspensions belonging to the experimental groups; and HDDA, resin, and photoinitiator were used as binders in a ratio of 7:3:0.1 for mixing all the powders. In the end, Disperbyk (BYK-180, BYK, USA) was added to all the suspensions. To ensure the homogeneous mixing of the suspensions, they were stirred in a vacuum for 200 s by using a planetary centrifugal mixer (ARV-310, Thinky Corp., Tokyo, Japan). The compositions of the prepared solutions are summarized in Table 2.

### 2.8. Observation of Colloidal Stability

The colloidal stability of the prepared zirconia suspensions was visually observed to determine their stability and the effect of the added dispersant. To this end, suspensions containing less than 1 vol.% of zirconia particles were used. Approximately, 10 mL of each suspension was poured into separate glass vials, and their stability was observed for up to 2 weeks (0, 5, 10, 15, and 20 days) until a clear sign was observed.

### 2.9. Viscosity

The viscosity values of the suspensions belonging to each group were measured using a viscometer (DV3T, Brookfield Engineering Laboratories, Stoughton, MA, USA). For this goal, various types of spindles exist and they were used based on the sample viscosities. The disk spindles produce accurate and reproducible results in the viscosity range of our suspensions.

To this end, the suspensions were placed at the center of a circular plate, and the distance between the plate and the spindle (CP4005, Brookfield Engineering Laboratories, Stoughton, MA, USA) was set to 2 mm. The viscosity was measured by changing the rotational speed of the cone at a constant temperature. Each group was measured at a shear rate of 1.2 to 20/s at 25 °C.

## 3. Results

### 3.1. Brunauer–Emmett–Teller Analysis

Brunauer–Emmett–Teller (BET) analysis was performed to determine the pore size and specific surface area of the selected zirconia particles (Table 3). Among the selected zirconia particles, the measured specific surface area and average pore size of ZS were 15.344 m^2^g^−1^ and 58.3 nm, respectively; those of ZM were 9.3932 m^2^g^−1^ and 31 nm; and those of ZL were 11.4 m^2^g^−1^ and 34.4 nm. These results confirmed that the total pore volume differed depending on the average pore size, which affected the specific surface area as well.

### 3.2. Particle Size Analysis and Zeta Analysis

**Figure 3:**
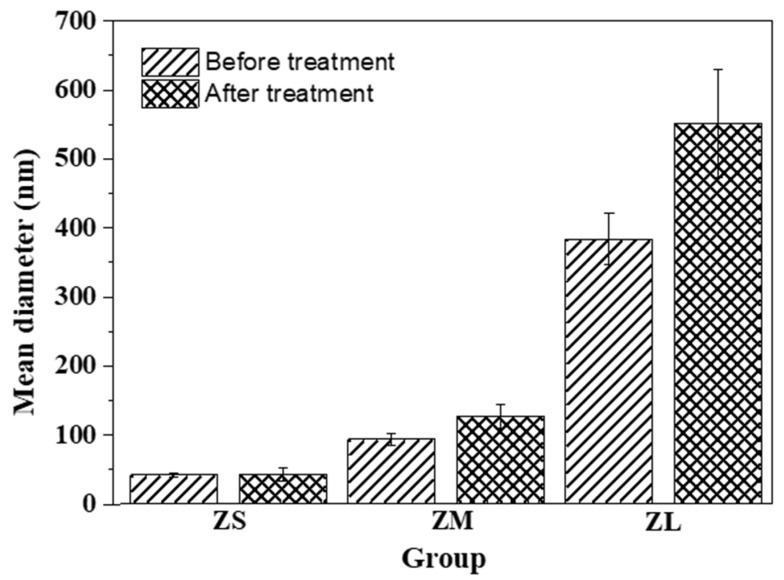
Size distribution of zirconia particles before and after silane coating.

**Figure 4:**
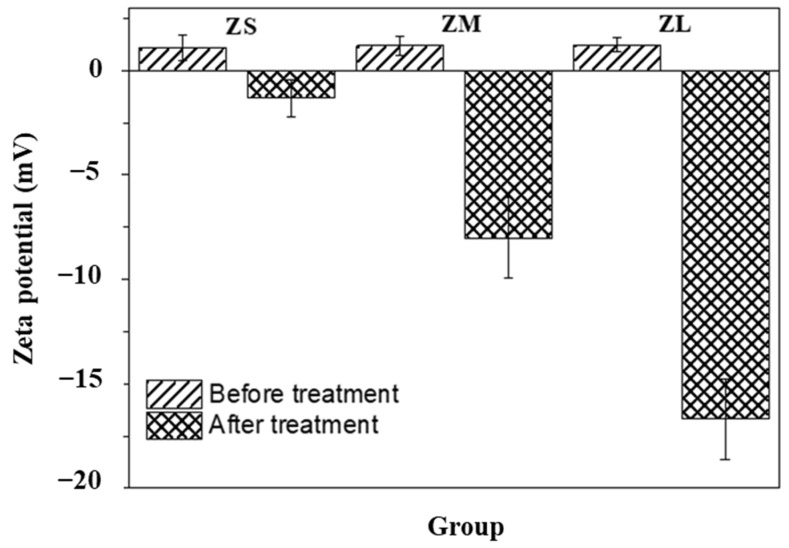
Zeta potentials of zirconia particles before and after silane coating.

Through dynamic light scattering, we measure and compare the particle size and surface charge of the zirconia particles. The respective results are demonstrated in Figure 3 and Figure 4.

### 3.3. Surface Treatment Analysis of Zirconia Particles

**Figure 5:**
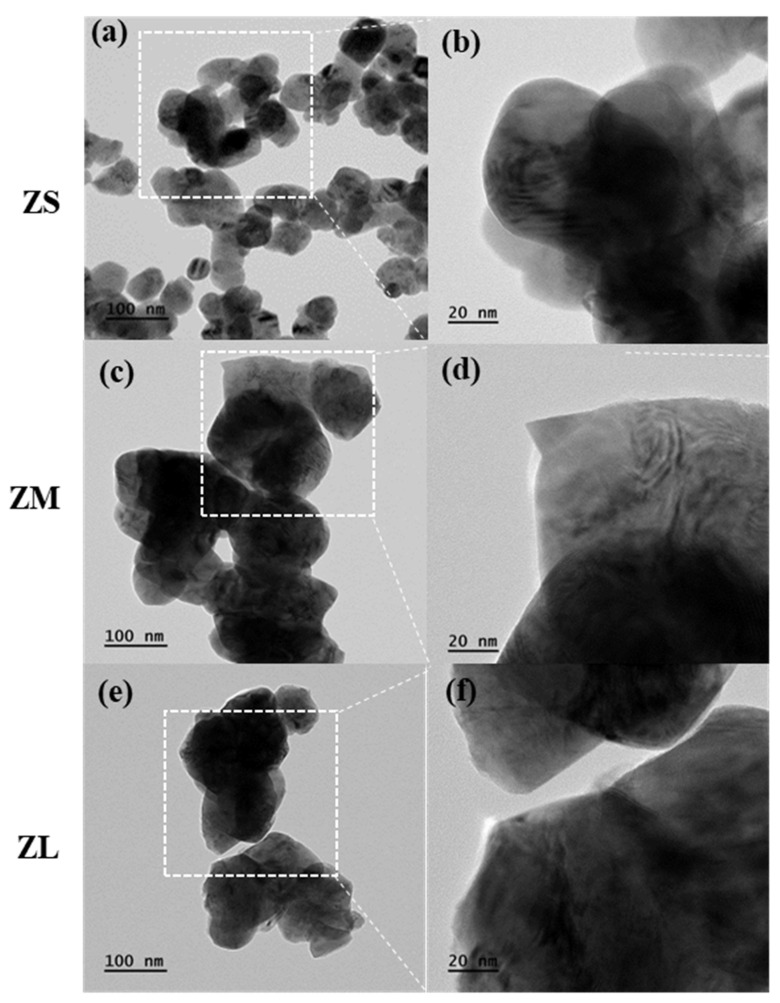
TEM images of control groups (ZS, ZM, ZL): (**a**) ZS ×100K, (**b**) ZS ×200K, (**c**) ZM ×100K, (**d**) ZM ×200K, (**e**) ZL ×100K, and (**f**) ZL ×200K.

**Figure 6:**
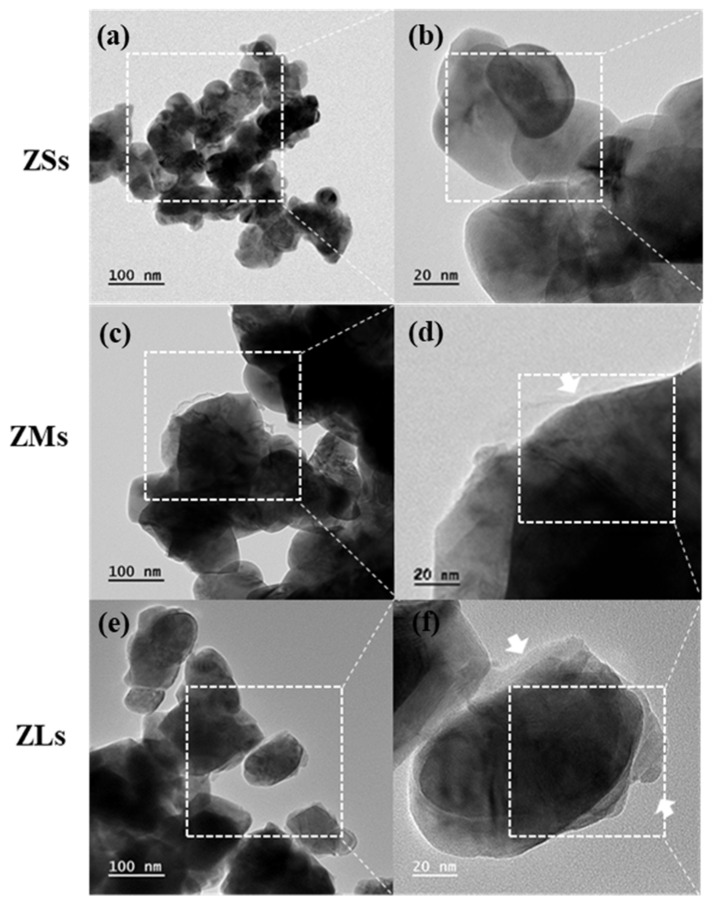
TEM images of experimental groups (ZSs, ZMs, ZLs): (**a**) ZSs ×100K, (**b**) ZSs ×200K, (**c**) ZMs ×100K, (**d**) ZMs ×200K, (**e**) ZLs ×100K, and (**f**) ZLs ×200K. Arrow indicate silane coating on the zirconia particle.

FE-TEM was used to identify the presence or absence of silanol coating on the control and experimental group particles, and depicted in the following Figure 5 and Figure 6.

### 3.4. FT-IR Analysis

**Figure 7:**
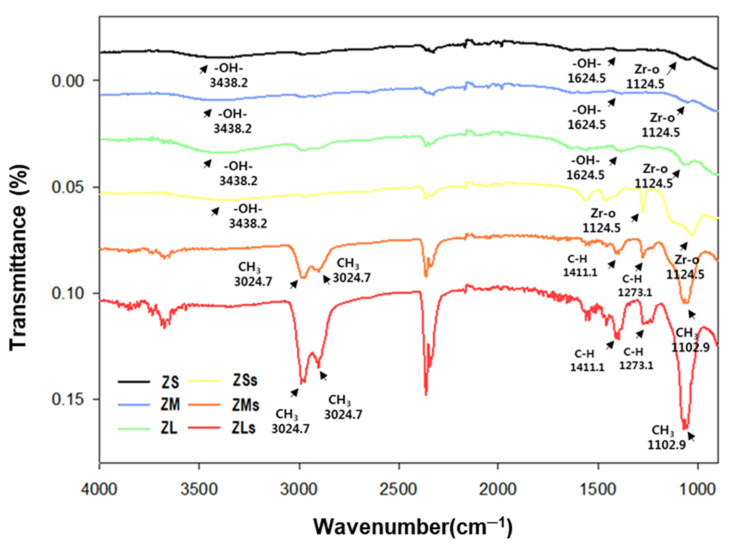
FT-IR spectra of various zirconia particles before and after silane coating.

To prove the effect of silane coating on the zirconia particles, the chemical reactions of the powders belonging to the control and experimental groups were analyzed by means of FT-IR spectroscopy (Figure 7).

### 3.5. Stability and Sedimentation Evaluation of Zirconia Suspensions

In the sedimentation test of the prepared zirconia suspensions belonging to both groups, they were visually evaluated from day 0 to day 20 to confirm their stability [5]. It was confirmed that the zirconia particles precipitated to different degrees over the set periods of 5, 10, 15, and 20 days. After 10 days, rapid particle precipitation was observed in the control groups.

**Figure 8:**
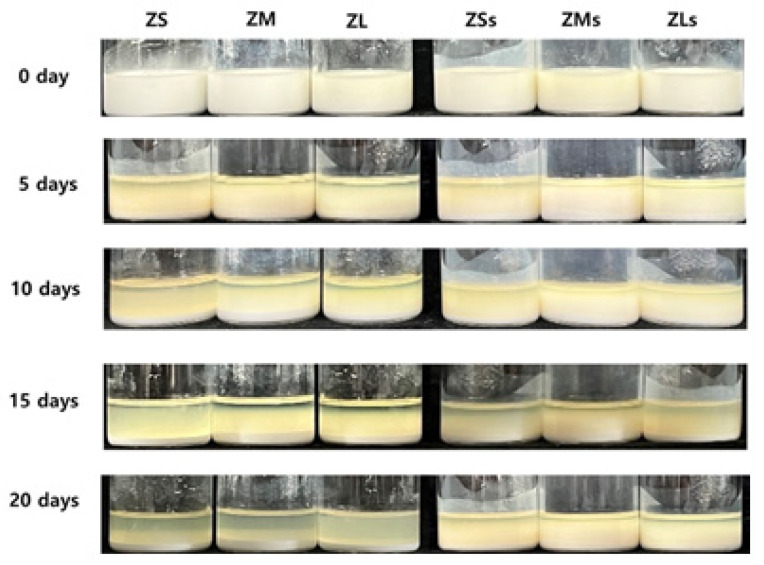
Sedimentation picture of suspensions with increasing days of before and after silane coating.

Moreover, rapid precipitation was observed in the experimental ZSs group. After 20 days, ZMs and ZLs remained as suspensions, and their dispersibility was maintained, as could be observed with the naked eye (Figure 8).

### 3.6. Viscosity

**Figure 9:**
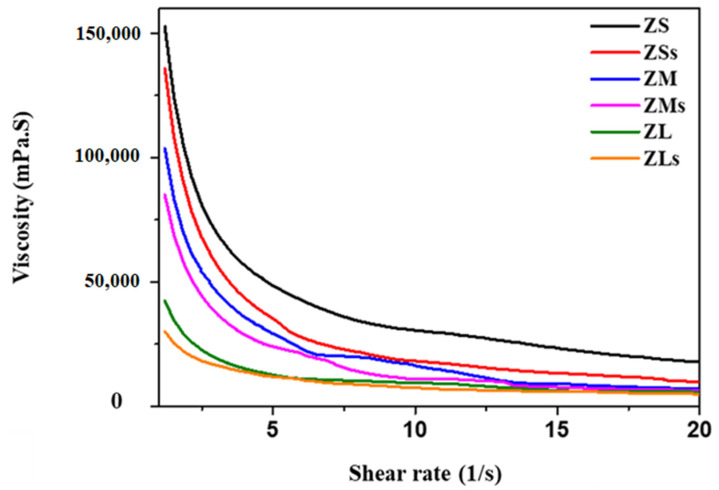
The viscosity of various zirconia suspensions before and after silane coating.

The viscosity of the zirconia suspensions belonging to the control and experimental groups were evaluated; and the rheological behaviors of the suspensions depending on the zirconia particle size, and the presence or absence of silane coating are depicted in Figure 9.

## 4. Discussion

The results of the Brunauer–Emmett–Teller (BET) analysis confirmed that the total pore volume differed depending on the average pore size, which affected the specific surface area as well. Particle size analysis was performed to confirm the change in particle size depending on whether the particles were subjected to surface treatment. In the case of the experimental groups (ZSs, ZMs, ZLs: 18.49 ± 0.14, 268.66 ± 17.35, 570.06 ± 570.24 nm, respectively), an increase in particle size was confirmed. In particular, the particle size of the ZLs group was the largest (Figure 3). By contrast, while ZSs had the smallest particle size among the experimental groups treated with silane, its particle size did not change significantly post-treatment. Owing to the high specific surface areas of ZM and ZL, their surfaces may not have been adequately coated with silane regarding the limited amount of it. It was confirmed that the organic coating of particles could be affected by various factors, such as particle size, pore size, pore area, and the amount of organic material used for coating. These factors should be adjusted and optimized depending on the characteristics of the particles [23,26,36].

The zeta potential measurements confirmed that the control groups exhibited weak anodic characteristics, while the zeta potential of the experimental groups changed to cathodic after silane treatment. This electrode reversal occurred because of the silanol treatment, and it was confirmed that the zeta potential value increased as the size of the particles in the experimental groups increased. The higher the absolute value of the repulsive electric force of a colloid suspended in a liquid, the higher the potential difference in the diffusion layer, which affects the uniform dispersion of the particles [37,38]. The ZLs group had the highest zeta potential value, and its viscosity and suspension stability was expected to improve because of the silanol treatment.

In the case of the ZSs group, the presence of a silane coating could not be confirmed. By contrast, in cases of the ZMs and ZLs groups, the presence of a silane coating was confirmed. In addition, we were able to confirm that the coating layer on the ZLs group particles was thicker because the number of particles and their specific surface area increased as the particle size decreased, even though the same quantities of zirconia particles and silane coupling agent were used across groups. The capacity of the limited silane coupling agent leads to differences in the coating thickness of the particles depending on the coating effect under various conditions (e.g., size, surface area, surface environment) [24,26].

To ensure effective silane coating of zirconia particles, it is necessary to determine the appropriate ratio of particle size to coating agent.

Among the results of the FR-IR spectroscopy of the silane-coated experimental groups, stretching vibrations of the characteristic C-H bands of silane appeared close to 1411.1 cm^−1^, 1273.1 cm^−1^, and 1102.9 cm^−1^ in the spectra of ZMs and ZLs. Moreover, –CH3 oscillations were observed at 3024.7 cm^−1^ and 2841.2 cm^−1^ [23,39]. Among the control groups, which were not coated with silane, only ZrO_2_-derived –OH bands at 3438.2 cm^−1^ and 1624 cm^−1^ and a Zr-O-derived band at 1124.5 cm^−1^ were observed [11]. The peaks at 1411.1 cm^−1^ and 1273.1 cm^−1^ were attributed to CH stretching (CH=CH2) due to the silane coating [40,41]. These results demonstrated that the silane coupling agent was coated on the zirconia particles.

The sedimentation test of the prepared zirconia suspensions confirmed that the zirconia particles precipitated to different degrees over the set periods of 5, 10, 15, and 20 days. After 10 days, rapid particle precipitation was observed in the control groups.

Moreover, rapid precipitation was observed in the experimental ZSs group. After 20 days, ZMs and ZLs remained as suspensions, and their dispersibility was maintained, as could be observed with the naked eye (Figure 8). This result could possibly be ascribed to the surface treatment of the ceramic particles, which increased their affinity toward the organic system of the photopolymerizable binder, and helped to maintain excellent particle dispersion and stability [42].

All the zirconia suspensions, prepared using a zirconia volume fraction of 40 vol.%, exhibited non-Newtonian shear-thinning behavior under shear rates of 1.2–20/s^−1^ (Figure 8). It was confirmed that the viscosity of these suspensions increased as the size of the added zirconia particles decreased. Compared to the viscosities of ZSs and ZLs, the viscosities of these suspensions decreased by 72.62–80.28% owing to the applied shear rates.

The viscosity values of the suspensions belonging to the experimental group were lower than those of the suspensions belong to the control group (Table 4).

This result was ascribed to the fact that the powders belonging to the experimental group reacted more effectively than those belonging to the control group and were better dispersed in the binder containing a dispersant that was involved in interparticle electrostatic interactions [43]. To realize uniform layer formation in the products manufactured using digital light processing (DLP), an additive manufacturing process, the suspension must have the appropriate viscosity characteristics and shear-thinning behavior to ensure that the photocuring mechanism and fluidity are maintained [44].

## 5. Conclusions

This study demonstrates the influence of zirconia particle size and the surface treatment using a silane coupling agent for the preparation of a suspension with optimal rheological properties. Based on the results, it is significant that the characteristics of candidate particles should be adequately analyzed before selecting them for use to ensure effective surface treatment and 3D additive manufacturing. In addition, a zirconia suspension with excellent features can be prepared by determining the appropriate ratio of coating agent suitable for the characteristics of the particles at hand. Although our experiments have a few limitations, for instance, different ratios of silane-coating agents were not used, we believe that the results of this study will act as a potential candidate in the field of ceramic additive manufacturing.

## Figures and Tables

**Table 1:** Zirconia particle sizes considered in this study.

Powder	Code	Commercial Size (nm)
TZ-3Y	ZS	40
TZ-3YS-E	ZM	90
TZY-3	ZL	300–600

**Table 2:** Experimental groups used in this study (vol.%).

Group (40 vol.%)		Zirconia	HDDA	Resin	Silane	Photo Initiator	Disperbyk	Total wt. (vol.%)
Control	ZS	40.00	39.54	15.47	0	0.50	4.48	100
	ZM	40.00	39.54	15.47	0	0.50	4.48	100
	ZL	40.00	39.54	15.47	0	0.50	4.48	100
Experiment	ZSs	40.00	35.93	14.06	5.07	0.45	4.48	100
	ZMs	40.00	35.93	14.06	5.07	0.45	4.48	100
	ZLs	40.00	35.93	14.06	5.07	0.45	4.48	100

**Table 3:** BET results of different powders.

Powder (Code)	Surface Area [m^2^/g]	Total Pore Volume (p/p^0^ = 0.990)	Mean Pore Diameter [nm]
TZ-3Y (ZS)	15.3	0.22	58.3
TZ-3YS-E (ZM)	9.4	0.05	31
TZY-3 (ZL)	11.4	0.1	33.4

**Table 4:** Change in viscosity with shear rate.

Shear Rate	ZS (mPa·s)	ZSs (mPa·s)	ZM (mPa·s)	ZMs (mPa·s)	ZL (mPa·s)	ZLs (mPa·s)
20 s^−1^	18,026	10,015	7047	6418	5789	4935
1.2 s^−1^	152,685	135,843	103,697	85,074	42,407	30,104

## Data Availability

All the data have been illustrated in the manuscript.
